# The Prognostic Value of FOXL2 Mutant Circulating Tumor DNA in Adult Granulosa Cell Tumor Patients

**DOI:** 10.3390/cancers17111894

**Published:** 2025-06-05

**Authors:** Geertruid J. Brink, Nizar Hami, Hans W. Nijman, Jurgen M. J. Piek, Luc R. C. W. van Lonkhuijzen, Eva Maria Roes, Ward Hofhuis, Christianne A. R. Lok, Cor D. de Kroon, Eelke H. Gort, Petronella O. Witteveen, Ronald P. Zweemer, Jolijn W. Groeneweg

**Affiliations:** 1 Department of Gynecologic Oncology, University Medical Center Utrecht, 3584 CX Utrecht, The Netherlands; 2 Department of Molecular Cancer Research, University Medical Center Utrecht, 3584 CX Utrecht, The Netherlands; 3 Department of Obstetrics and Gynecology, University Medical Center Groningen, 9713 GZ Groningen, The Netherlands; 4 Department of Obstetrics and Gynecology, Catharina Hospital, 5623 EJ Eindhoven, The Netherlands; 5 Center for Gynecologic Oncology Amsterdam, Cancer Center Amsterdam, Amsterdam University Medical Center, 1105 AZ Amsterdam, The Netherlands; 6 Department of Gynecologic Oncology, Erasmus MC Cancer Institute, 3015 GD Rotterdam, The Netherlands; 7 Department of Obstetrics and Gynecology, Franciscus Gasthuis en Vlietland, 3045 PM Rotterdam, The Netherlands; 8 Department of Gynecological Oncology, Center Gynaecologic Oncology Amsterdam, 1066 CX Amsterdam, The Netherlands; 9 Department of Gynecology, Leiden University Medical Center, 2333 ZG Leiden, The Netherlands; 10 Department of Medical Oncology, University Medical Center Utrecht, 3584 CX Utrecht, The Netherlands

**Keywords:** granulosa cell tumor, ovarian cancer, circulating tumor DNA, prognostic, diagnostic, FOXL2

## Abstract

**Objectives:** The purpose of the study is to determine whether *FOXL2* circulating tumor DNA can be used as a prognostic biomarker and marker for monitoring treatment response in patients with an adult-type granulosa cell tumor (aGCT). **Methods:** Plasma samples of patients included in the multicenter GRANULOSA study were collected before and after surgery for primary or recurrent aGCT, during follow-up, and during systemic treatment. The presence of ctDNA containing the *FOXL2 402C>G* mutation was analyzed in 284 samples from 20 primary and 34 recurrent aGCT patients, using digital droplet PCR. Clinical data were retrieved from electronic patient records, and patients were followed through January 2025. **Results:** *FOXL2* mutant ctDNA was detected in 28 of 54 patients (48%). In primary aGCT, recurrences were more frequently seen in patients with detectable ctDNA (33% vs. 18%), and ctDNA remained detectable postoperatively in some cases despite complete cytoreduction. In recurrent aGCT patients, detectable ctDNA was associated with significantly worse overall survival (*p* = 0.023), and the postoperative presence of ctDNA following complete debulking surgery was significantly associated with a shorter recurrence-free survival (4.7 vs. 11.6 months, *p* = 0.025). **Conclusions:** *FOXL2* mutant ctDNA could be a prognostic biomarker in aGCT, being associated with worse overall survival in recurrent aGCT patients. In addition, the presence of ctDNA after surgery could reflect the presence of minimal residual disease, negatively impacting the disease course. The implementation of *FOXL2* ctDNA measurement in clinical practice may help to identify high-risk aGCT patients.

## Simple Summary

Adult-type granulosa cell tumor is a rare form of ovarian cancer that can come back years after initial treatment. Current blood tests are not always reliable for detecting the disease or predicting how it will progress. In this study, we investigated whether a small fragment of tumor DNA found in the blood—called circulating tumor DNA (ctDNA)—could help identify patients at higher risk of their cancer returning or not responding well to treatment. We detected this ctDNA using a specific genetic alteration, the *FOXL2* mutation, which is common in this tumor type. Our results show that patients with detectable ctDNA often have worse outcomes, even after surgery. This suggests that ctDNA analysis could help doctors predict which patients need closer monitoring or additional treatment, potentially improving long-term care for people with this rare cancer.

## 1. Introduction

Adult-type granulosa cell tumor (aGCT) is a rare subtype of ovarian cancer, with a worldwide incidence estimated at 1.1 per 100,000 women [1]. Tumor tissue of aGCT patients exhibits a *FOXL2 402C>G* mutation in 97%; it is a unique characteristic of this tumor type [2]. Clinically, aGCT patients often present with abdominal pain and/or vaginal bleeding, the latter due to estradiol production by the tumor [3]. After primary surgical treatment, approximately one third of patients develop a recurrence. Recurrences typically occur late, which is why women with aGCT are often followed for extensive periods of time. Surgery remains the cornerstone of treatment for both primary tumors and recurrent disease. When surgery is not feasible, systemic therapies, including chemotherapy and anti-hormonal treatments, are used.

In addition to estradiol, inhibin B, inhibin A, and anti-Müllerian hormone (AMH) are produced by granulosa cells and are often elevated in aGCT. These hormones are, therefore, used as tumor markers, with increased inhibin B and AMH being the most accurate tumor markers for aGCT [4]. Although their reported sensitivity rates are as high as 90%, levels can fluctuate over time and do not always correlate with disease activity [5,6,7]. Especially in premenopausal women, physiological elevation and fluctuations in hormone levels are often seen, causing concerns in aGCT patients during follow-up. Inhibin B is the most commonly used and extensively studied marker for monitoring aGCT [8,9,10,11]. However, as it primarily reflects tumor load rather than tumor behavior, its prognostic value remains unestablished.

Recent developments have highlighted the potential of circulating tumor DNA (ctDNA) as a non-invasive marker for cancer detection and monitoring in a variety of cancers, including ovarian cancer [12,13,14]. In addition, studies have demonstrated the use of ctDNA to evaluate treatment response and detect minimal residual disease, underscoring its potential prognostic and predictive value in breast cancer and gynecologic malignancies [13,14,15,16,17].

Previous studies have identified ctDNA harboring the *FOXL2 402C>G* mutation in the plasma of patients with aGCT [18,19]. Research by our group has shown the presence of *FOXL2* mutant ctDNA in the majority of aGCT patients and suggested a correlation between *FOXL2* mutant ctDNA levels and disease activity in a subset of patients [19]. To date, the prognostic value of ctDNA in aGCT has not been investigated.

Therefore, the aim of this study is to determine the prevalence of *FOXL2* mutant ctDNA in prospectively collected plasma samples from a large cohort of aGCT patients to establish its use as a prognostic and predictive biomarker for future clinical use.

## 2. Materials and Methods

### 2.1. Samples and Patients

Plasma samples were prospectively collected from aGCT patients as part of the multicenter GRANULOSA study [20]. Ethical approval was obtained by the Medical Ethics Committee of the UMC Utrecht (UMCU METC 17-868). Patients diagnosed with primary or recurrent aGCT were included and all provided written informed consent.

Blood samples were collected between June 2018 and May 2024. Samples were taken preoperatively, postoperatively, during follow-up, and when systemic treatment was administered. Postoperative samples were taken within three months of the operation (range 2–13 weeks). Samples during systemic treatment were collected prior to each chemotherapy cycle or every three months during anti-hormonal therapy. If possible, blood samples were combined with routine serum marker inhibin B measurements. Clinical data were retrieved from electronic patient records. Patients were followed until January 2025. We refrained from testing for the presence of the *FOXL2 c.402C>G* mutation in tumor tissue, as this mutation is known to be present in 97% of aGCT based on our own prior study confirming its presence in nearly all cases [2,19].

Recurrence-free survival (RFS) was defined as the time between the date of surgery and the first sign of recurrent disease on imaging, often following an increase in inhibin B. Overall survival (OS) was defined as the time between the date of surgery for primary diagnosis and end of follow-up or death. Treatment response was based on imaging and defined according to the RECIST criteria: complete response (CR), partial response (PR), stable disease (SD), and progressive disease (PD) [21]. The status of the disease was categorized as no evidence of disease (NED), alive with disease (AWD), died of disease (DOD), and died of other causes (DOC).

### 2.2. Sample Preparation and Digital Droplet PCR

Blood withdrawal and sample preparation have been previously described, and the full protocol experiment methods can be found in Appendix A [19]. In short, venous blood samples were withdrawn in two 10 mL PAXgene blood tubes (BD Biosciences, Eysins, Switzerland). Within 7 days after collection, samples were centrifuged, and supernatant plasma was stored in aliquots at −80 °C until further analysis.

Plasma aliquots were thawed, and approximately 3mL plasma per sample was used for cell-free DNA (cfDNA) isolation using the QIAamp Circulating Nucleic Acid Kit (Qiagen, Hilden, Germany). Quantification of isolated cfDNA was performed using the Qubit fluorometer with the dsDNA High Sensitivity Assay Kit (Thermo Fisher Scientific, Waltham, MA, USA), and quality control was performed using the Agilent TapeStation system with D5000 ScreenTape assay (Agilent, Santa Clara, CA, USA).

The cell-free DNA extracted from plasma samples was analyzed for mutant *FOXL2* by digital droplet PCR (ddPCR). The ddPCR mutation assays for *FOXL2* wild-type (WT) and *FOXL2 c.402C > G* p.C134W from Bio-Rad Laboratories (Hercules, CA, USA) were used. The thermal cycling conditions for the FOXL2 assay were 95 °C for 10 min, followed by 40 cycles of 95 °C for 30 s and 55 °C for 1 min, followed by 98 °C for 10 min and infinite hold at 12 °C. Positive and negative controls, including aGCT tumor DNA samples with and without the *FOXL2* mutation, as well as no-template controls, were incorporated into every assay run. Each cfDNA sample was evaluated in duplicate wells at a minimum for each run and underwent a minimum of two independent ddPCR runs.

### 2.3. Data and Statistical Analysis

As described before, ddPCR data were processed using Quantasoft software version 1.7 (Bio-Rad Laboratories) [19]. Only wells containing more than 10,000 total droplets were included in the analysis. For each ddPCR assay, manual threshold settings were applied to distinguish positive from negative droplets based on the distribution observed in the positive and negative control samples. Double-positive droplets were excluded. Samples with two or more mutation-positive droplets per well were considered true positive. The relative concentration of ctDNA in each plasma sample was quantified by calculating the fractional abundance, expressed as the percentage of mutant to total (mutant + wild − type) copies.

Statistical analyses were conducted using SPSS Statistics for Windows, Version 30.0.0 (IBM Corp., Armonk, NY, USA), and GraphPad Prism version 10.4.1 for Windows (GraphPad Software, Boston, MA, USA). Descriptive statistics were applied, with non-normally distributed data presented as medians with interquartile ranges (IQRs). Group comparisons were performed using the Mann–Whitney U test, and survival analyses were conducted using the Kaplan–Meier method and the log-rank test.

## 3. Results

Analysis of *FOXL2* mutant ctDNA was performed in 332 samples from 79 patients. A subset of patients (n = 25) was included during routine follow-up without clinical signs of disease and without the development of recurrence during subsequent follow-up. Because the absence of *FOXL2* mutant ctDNA in these samples could reflect either the true absence of disease or non-detectable ctDNA, these patients (48 samples) were excluded from further analysis.

In 28 (48%) out of 54 patients, *FOXL2* mutant ctDNA was detected, and in the remaining 26 patients (52%), ctDNA was undetectable. Baseline characteristics of 54 patients are summarized in Table 1. Among patients with detectable ctDNA, the fractional abundance varied greatly (median 1.34%, IQR: 0.33–9.00). While a large proportion of patients had a low fractional abundance in all samples, 12 patients (43%) with detectable ctDNA had a fractional abundance greater than five percent.

**Figure 1:**
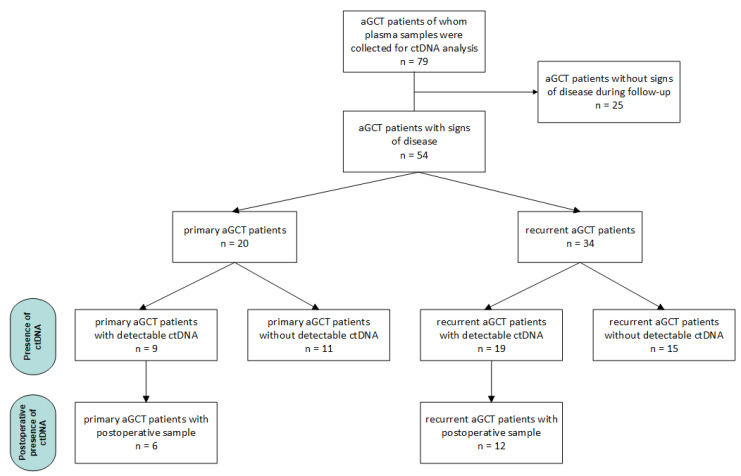
Flowchart of patient selection and allocation for *FOXL2* mutant ctDNA analyses in aGCT.

Subsequent analyses on the prognostic and predictive value of *FOXL2* mutant ctDNA were performed in primary and recurrent aGCT patients separately. In a subset of primary and recurrent aGCT patients, postoperative samples were available for ctDNA analysis. Figure 1 provides an overview of all patients and their allocation to specific analyses based on disease status and the moment of sampling.

### 3.1. Primary aGCT Patients

#### 3.1.1. Prognostic Value of the Presence of ctDNA

In 20 primary aGCT patients, the presence of *FOXL2* mutant ctDNA as a possible prognostic factor for the development of recurrence was investigated. The median duration of follow-up was 2.5 years (IQR: 1.3–4.0 years). In nine primary aGCT patients (45%), ctDNA was detected, and eleven patients (55%) had no measurable ctDNA. In total, five patients developed a recurrence: three in the group with detectable ctDNA (33.3%) and two in the group without detectable ctDNA (18.2%). Due to the small sample size and short follow-up time, these findings are descriptive, and statistical testing could not be performed.

#### 3.1.2. Postoperative Detection of ctDNA

In six of nine primary aGCT patients with detectable *FOXL2* mutant ctDNA, postoperative plasma samples were available for ctDNA assessment. In all patients, primary surgery was performed with complete resection of the tumor and no visible residual disease. The median duration of follow-up was 2.8 years (IQR: 0.96–4.7 years). In three patients, no ctDNA could be detected postoperatively (see Table 2), and none of them developed a recurrence during follow-up. In three patients, low levels of *FOXL2* mutant ctDNA were found postoperatively. The patient with the highest postoperative fractional abundance developed a recurrence after six months. In another patient with a fractional abundance of 0.05%, a recurrence was found after 3.6 years. In another patient, a postoperative fractional abundance of 0.12% was found; however, she did not develop a recurrence during a six-year follow-up. Given the small number of patients and limited follow-up, this analysis is descriptive in nature, and no statistical testing could be performed.

In four of six patients, inhibin B was determined in the same week as ctDNA. In all four patients, including all three patients in whom ctDNA was detected postoperatively, postoperative inhibin B was normal, as shown in Table 2.

### 3.2. Recurrent aGCT Patients

#### 3.2.1. Prognostic Value of the Presence of ctDNA

**Figure 2:**
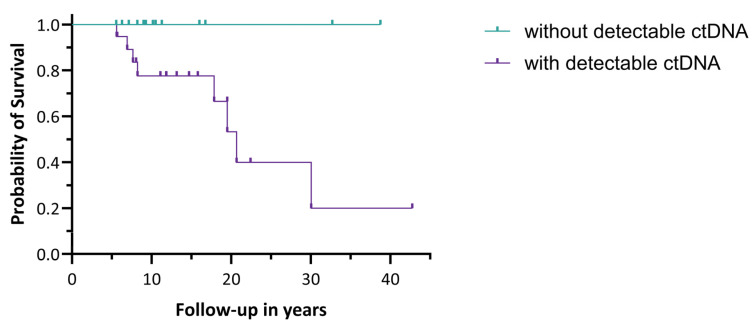
Overall survival in recurrent aGCT patients stratified by their ctDNA status. The log-rank test showed a significant difference in OS between the two groups (*p* = 0.023).

In 34 patients with recurrent aGCT, the presence of *FOXL2* mutant ctDNA as a possible prognostic factor for OS was investigated. The median duration of follow-up was 11.2 years (IQR: 8.2–18.3 years). Nineteen of the thirty-four recurrent aGCT patients (56%) had detectable ctDNA levels, and in fifteen patients (44%), no ctDNA was detected based on samples collected at various time points during the disease course. In the group with detectable ctDNA, eight patients died of aGCT, while no patients died in the group without detectable ctDNA, as shown in Figure 2. The log-rank test showed a significant difference in OS between the two groups (*p* = 0.023). No significant difference in the median duration of follow-up was observed between the two groups. All eight deceased patients had a relatively high fractional abundance (median 13.58%, IQR: 2.93–19.75).

#### 3.2.2. Postoperative Detection of ctDNA

In 12 of 19 patients with recurrent aGCT and detectable ctDNA, complete debulking surgery was performed, and postoperative plasma samples were collected and assessed for the presence of ctDNA. The median duration of follow-up was 12.9 years (IQR: 9.0–17.7 years). In nine patients, low levels of *FOXL2* mutant ctDNA were found postoperatively, and eight of these patients developed a new recurrence during subsequent follow-up. In three patients, ctDNA was undetectable postoperatively, and despite this, all three patients developed a new recurrence. In all patients, inhibin B was measured in the same week, and its levels varied greatly, as shown in Table 3. The median time until the next recurrence was significantly shorter in patients with detectable ctDNA postoperatively compared to those without detectable postoperative ctDNA: 4.7 months (IQR: 3.6–6.0 months) and 11.6 months (range: 8.5–37.8 months), respectively (*p* = 0.025).

### 3.3. ctDNA as a Biomarker for Monitoring Systemic Therapy

**Figure 3:**
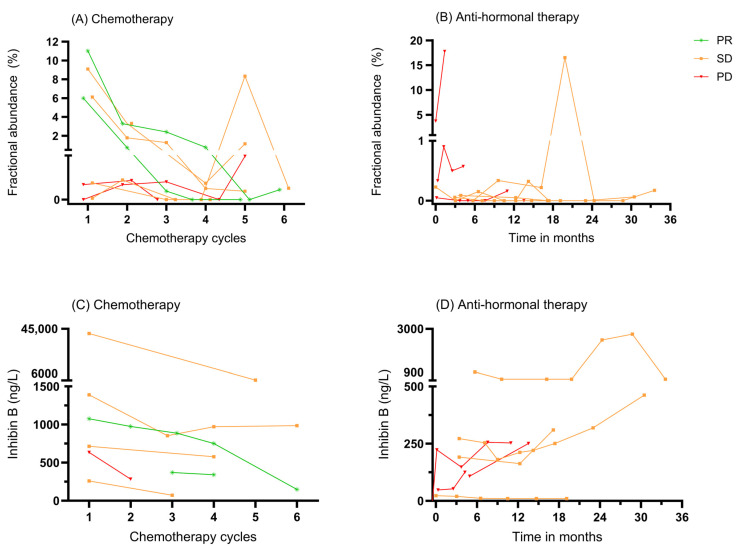
(**A**) The course of fractional abundance per chemotherapy cycle, linked to response according to the RECIST criteria. (**B**) The course of fractional abundance over time during anti-hormonal therapy, linked to response according to the RECIST criteria. (**C**) The course of inhibin B per chemotherapy cycle, linked to response according to the RECIST criteria. (**D**) The course of inhibin B over time during anti-hormonal therapy, linked to response according to the RECIST criteria. PD: progressive disease, PR: partial response, SD: stable disease.

Several recurrent aGCT patients were treated with systemic therapy, and we investigated if systemic treatment responses could be monitored with ctDNA. Eight patients were treated with chemotherapy, and samples were taken with each course. Two patients had a PR, four patients had SD, and two patients had PD, as shown in Figure 3A. Both patients with a PR showed an ongoing decline in fractional abundance with each cycle given, decreasing to 0% ctDNA measured by the end of six cycles. Patients with SD or PD displayed a more variable pattern, with some patients showing an initial decline, followed by fluctuations or even increases.

Eight patients were treated with anti-hormonal therapy for different durations. Four patients achieved SD and four patients had PD, as shown in Figure 3B. The ctDNA fractional abundance in patients with PD receiving anti-hormonal therapy showed a clear increase. In patients with SD, fluctuating ctDNA levels were observed.

In comparison, the course of inhibin B initially showed a decline during chemotherapy in all cases, regardless of the final response, as shown in Figure 3C. In one of two patients who achieved a PR, a clear decrease in inhibin B was observed as chemotherapy cycles progressed. During anti-hormonal therapy, an increase in inhibin B levels was seen in almost all patients, including those with PD and SD, as shown in Figure 3D.

## 4. Discussion

The prognostic value of ctDNA has not been previously investigated in aGCT. After our initial investigation describing the use of ctDNA harboring the *FOXL2 402C>G* mutation as a biomarker in aGCT, the purpose of the current study was, therefore, to determine its prognostic and predictive utility [19].

In patients with primary aGCT, the presence of *FOXL2* mutant ctDNA showed a suggestion of a higher recurrence risk as compared to patients with non-detectable ctDNA. This trend is consistent with studies in other cancers, demonstrating an association between the presence of ctDNA and the risk of recurrence [14,17,22]. However, our findings must be interpreted with caution due to several important limitations. First, the sample size of this subgroup is small (n = 20). Second, the median follow-up duration of 2.5 years is relatively short, especially for a disease such as aGCT, which is known for its potential for very late recurrences. Further, prospective research in larger cohorts is essential before any conclusion on clinical implications can be drawn.

In patients with recurrent disease, significantly worse overall survival was found in patients with detectable ctDNA. This is consistent with studies in other malignancies, including epithelial ovarian cancer, where Heo et al. identified ctDNA as a highly predictive marker for survival [23]. In rectal cancer, several studies have shown that the presence of ctDNA at baseline is associated with worse OS [24]. In small cell lung cancer, it was demonstrated that ctDNA is an important predictor of poor OS [25]. A study in colorectal cancer found that in patients with recurrent disease, ctDNA positivity correlated with shorter OS [26]. In a large meta-analysis of breast cancer, a significant association between ctDNA detection and worse survival was revealed [27]. It remains unknown whether adjusting treatment strategies based on the detection of ctDNA would influence prognosis, which could also be relevant for aGCT.

The observed presence of *FOXL2* mutant ctDNA after complete surgical resection in a subset of patients is another novel finding in aGCT. In epithelial ovarian cancer as well as other malignancies, the detection of ctDNA following (most often surgical) treatment has been described as the presence of minimal residual disease [28,29,30]. Supporting the suggestion that postoperative ctDNA signifies minimal residual disease, a significantly shorter RFS was observed among recurrent aGCT patients who were found to harbor ctDNA after a complete debulking surgery (4.7 vs. 11.6 months). These results are in line with numerous other studies in other malignancies, including breast and epithelial ovarian cancer [16]. Hou et al. showed a reduced RFS in epithelial ovarian cancer patients with the presence of ctDNA postoperatively [15]. Recently, these results were confirmed by Kallio et al. and Shu et al., showing high sensitivity and specificity of ctDNA analysis in detecting postoperative minimal residual disease and a shorter RFS in patients with detectable ctDNA postoperatively [31,32]. These findings also seem to apply to our patients with non-epithelial ovarian cancer.

Of interest, in postoperative plasma samples of primary aGCT patients with the presence of *FOXL2* mutant ctDNA, normal values of inhibin B were seen. This may suggest a potential benefit of ctDNA in detecting minimal residual disease. This pilot observation parallels findings in epithelial ovarian cancer, where ctDNA has been shown to outperform the regular marker CA-125 in predicting recurrence [15,33]. This finding requires further confirmation.

Our findings suggest that *FOXL2* mutant ctDNA could serve as a prognostic biomarker in aGCT, with potential clinical relevance by identifying patients at higher risk of recurrence and worse survival. This may help to guide aGCT management. The analysis of *FOXL2* mutant ctDNA could facilitate a more patient-tailored approach, such as intensified monitoring or consideration of additional therapy when ctDNA is present.

In patients treated with chemotherapy who started with a high fractional abundance of *FOXL2* mutant ctDNA, a rapid decrease in ctDNA levels was clearly observed in the case of a PR and in some patients with SD. Conversely, a less pronounced response or a low initial fractional abundance led to more variation in ctDNA levels, making interpretation less reliable. This was also seen in patients treated with anti-hormonal therapy. Compared to inhibin B, ctDNA appears to better distinguish treatment response during systemic therapy, particularly in cases with a high initial fractional abundance. These findings highlight the potential use of ctDNA for monitoring treatment response, particularly in patients with a high initial fractional abundance. This aligns with Wyatt et al., who evaluated the potential of ctDNA as an early biomarker for treatment response in metastatic cancers, highlighting its correlation with RECIST criteria and potentially allowing for more timely adjustments in therapy [34].

As Krebs et al. insightfully displayed, ctDNA analysis can be used for diagnosis, prognosis, intervention outcome monitoring by earlier detecting residual disease or recurrence, and treatment response monitoring [13]. While ctDNA shows great potential, its routine clinical implementation is challenged by economic feasibility and workflow variability, as highlighted by Kramer et al. [35,36].

The strengths of this study include its prospective design and the unique applicability of *FOXL2* mutant ctDNA as a biomarker, given that nearly all aGCTs harbor this mutation. Additionally, the study provides a direct comparison between ctDNA levels and clinical outcomes, offering valuable insights into the prognostic utility of ctDNA. Limitations of this study include the inherent bias in the cohort, as it contains a higher proportion of patients with recurrent disease. However, this has allowed us to investigate recurrent aGCT and overcome the commonly long interval between primary and recurrent aGCT. To minimize the impact of this bias, analyses for primary and recurrent patients were performed separately. Other limitations include the small sample size, particularly of the primary aGCT subgroup, the relatively short follow-up periods, and the lack of uniformity in sampling times due to the multicenter design. These limitations reflect the realities of clinical practice, where creating perfectly controlled conditions is often not feasible. In addition, it is important to emphasize that aGCT is an extremely rare cancer, and this multicenter research resulted in a large cohort of patients and plasma samples.

## 5. Conclusions

With this pilot study investigating the prognostic value of *FOXL2* mutant ctDNA in aGCT, it was shown that ctDNA presence may negatively impact prognosis. The addition of ctDNA analysis in the management of aGCT could help to identify patients with a higher risk of recurrence and a worse prognosis. Further research is warranted to determine the accuracy of *FOXL2* mutant ctDNA as a prognostic marker in aGCT.

## Figures and Tables

**Table 1:** Baseline characteristics of the total cohort and the subgroups with detectable ctDNA and non-detectable ctDNA. Data are presented as n (%) or median (IQR). Follow-up is defined as the time from diagnosis until the last recorded follow-up in January 2025. Disease status was assessed at the time of the last follow-up.

Characteristics	Total	Detectable ctDNA	Non-Detectable ctDNA	*p*-Value
Patients	54	28	26	
Year of diagnosis	2015 (2010–2021)	2013 (2006–2018)	2017 (2014–2021)	0.046
Age at diagnosis in years	49 (42–62)	49 (44–56)	50 (39–67)	0.808
Tumor stage at diagnosis				0.610
IA	23 (43)	11 (39)	12 (46)	
IB	1 (2)	1 (4)	0 (0)	
IC	26 (48)	15 (53)	11 (42)	
II	3 (5)	1 (4)	2 (8)	
III	1 (2)	0 (0)	1 (4)	
Follow-up time in years	8.2 (3.1–15.0)	10.0 (5.6–17.7)	6.7 (2.1–10.5)	0.087
Recurrence				0.366
Yes	39 (72)	22 (79)	17 (65)	
No	15 (28)	6 (21)	9 (35)	
Disease status				0.005
No evidence of disease	19 (35)	7 (25)	12 (46)	
Alive with disease	26 (48)	12 (43)	14 (54)	
Dead of disease	9 (17)	9 (32)	0 (0)	
Highest fractional abundance		1.34 (0.33–9.00)		

**Table 2:** Fractional abundance and inhibin B in the pre- and postoperative samples of six primary aGCT patients with detectable ctDNA.

Patient ID	Weeks After Surgery	Preop FA%	Postop FA%	Inhibin B (ng/L)	Recurrence During Follow-Up	Duration FU (Years)
P1	7	n/a	0.12	10	−	6
P2	4	7.90	0.44	10	+	1
P3	3	n/a	0.05	10	+	4
P4	7	0.92	0.00	10	−	3
P5	2	8.74	0.00	n/a	−	3
P6	5	0.26	0.00	n/a	−	1

FA: fractional abundance; FU: follow-up; n/a: not available; postop: postoperative; preop: preoperative.

**Table 3:** Determination of fractional abundance and inhibin B in the pre- and postoperative samples of 12 recurrent aGCT patients with detectable ctDNA.

Patient ID	Number of Recurrences	Weeks After Surgery	Preop FA%	Postop FA%	Inhibin B (ng/L)	New Recurrence During Follow-Up
P7	3	7	18.1	0.70	62	+
P8	3	7	0.15	1.86	84	+
P9 *	4	13	7.54	0.15	87	−
P10	5	7	n/a	0.06	101	+
P11	2	13	0.22	0.00	10	+
P12	1	10	n/a	0.00	133	+
P13	4	6	0.00	0.11	246	+
P14	2	7	n/a	0.06	11	+
P15	2	7	1.44	0.22	25	+
P16	4	6	n/a	0.00	208	+
P17	1	9	0.02	0.12	10	+
P18	1	5	0.43	0.23	833	+

* Follow-up after this postoperative sample was 2.2 years. FA: fractional abundance, n/a: not available, postop: postoperative, preop: preoperative.

## Data Availability

The data presented in this study are available upon request from the corresponding author. The data are not publicly available due to privacy concerns involving sensitive patient information and the ongoing nature of the clinical study. Data sharing is limited to safeguard participant confidentiality and in compliance with institutional ethical guidelines.

## Abbreviations

The following abbreviations are used in this manuscript:

aGCT	Adult-type granulosa cell tumor
AMH	Anti-Müllerian hormone
AWD	Alive with disease
cfDNA	Cell-free DNA
CR	Complete response
ctDNA	Circulating tumor DNA
ddPCR	Digital droplet PCR
DOC	Died of other causes
DOD	Died of disease
IQR	Interquartile range
NED	No evidence of disease
OS	Overall survival
PD	Progressive disease
PR	Partial response
RFS	Recurrence-free survival
SD	Stable disease

## Appendix A. A Detailed Protocol of Sample Preparation and ddPCR Analysis

Two 10 mL PAXgene blood tubes were venously withdrawn (BD Biosciences, Eysins, Switzerland). Within 7 days after collection, samples were centrifuged for 10 min at 1900× *g* (3000 rpm) at 4 °C. The supernatant plasma was then transferred to 15 mL centrifuge tubes and centrifuged for 10 min at 16,000× *g* (in a fixed-angle rotor) to remove additional cellular nucleic acids attached to cell debris. The supernatant was divided into 1 ml aliquots and stored at −80 °C until further analysis.

For the isolation of cell-free DNA, plasma aliquots were thawed and per sample; approximately 3 mL of plasma was isolated using the QIAamp Circulating Nucleic Acid Kit (Qiagen, Hilden, Germany) following the manufacturer’s instructions. Isolated DNA samples were eluted in a 35 μL elution buffer. Quantification of isolated DNA samples and quality control was measured using a Qubit fluorometer with the dsDNA High Sensitivity Assay Kit (Thermo Fisher Scientific, Waltham, MA, USA) and Agilent TapeStation system with the D5000 ScreenTape assay (Agilent, Santa Clara, CA, USA), respectively.

The cell-free DNA extracted from plasma samples was analyzed for mutant FOXL2 by digital droplet PCR (ddPCR). The ddPCR mutation assays for FOXL2 wild-type (WT) and *FOXL2 c.402C > G* p.C134W from Bio-Rad Laboratories (Hercules, CA, USA) were used. Reaction volumes of 22 μL per well of a 96-well plate were prepared. Each reaction for *FOXL2* testing contained 11 μL supermix for probes (no dUTP) (BioRad Laboratories), 1 μL primer–probe mix for both mutants (labeled with FAM), WT (labeled with HEX) *FOXL2*, 4–8 μL cfDNA from patient plasma, and purified water to a total of 22 μL. Reactions were subjected to ddPCR analysis using the QX200 system according to the manufacturer’s protocol (Bio-Rad Laboratories). The thermal cycling conditions for the *FOXL2* assay were 95 °C for 10 min, followed by 40 cycles of 95 °C for 30 s and 55 °C for 1 min, followed by 98 °C for 10 min and infinite hold at 12 °C. Positive and negative controls, consisting of aGCT tumor DNA samples with and without the *FOXL2* mutation, as well as no-template controls, were included in every run. Each cfDNA sample was analyzed at least in duplicate wells in each run and in at least two separate ddPCR runs.
